# Screening for Potential Antiviral Compounds from Cyanobacterial Secondary Metabolites Using Machine Learning

**DOI:** 10.3390/md22110501

**Published:** 2024-11-05

**Authors:** Tingrui Zhang, Geyao Sun, Xueyu Cheng, Cheng Cao, Zhonghua Cai, Jin Zhou

**Affiliations:** 1 Marine Ecology and Human Factors Assessment Technical Innovation Center of Natural Resources Ministry, Tsinghua Shenzhen International Graduate School, Shenzhen 518055, China; zhangtr23@mails.tsinghua.edu.cn (T.Z.); caizh@sz.tsinghua.edu.cn (Z.C.); 2 Shenzhen Public Platform for Screening and Application of Marine Microbial Resources, Institute for Ocean Engineering, Shenzhen International Graduate School, Tsinghua University, Shenzhen 518055, China; 3 Shenzhen Key Laboratory of Advanced Technology for Marine Ecology, Institute for Ocean Engineering, Shenzhen International Graduate School, Tsinghua University, Shenzhen 518055, China; 4 Institute for Ocean Engineering, Shenzhen International Graduate School, Tsinghua University, Shenzhen 518055, China

**Keywords:** cyanobacterial secondary metabolites, anti-viral compounds, machine learning, molecular docking, application potential

## Abstract

The secondary metabolites of seawater and freshwater blue-green algae are a rich natural product pool containing diverse compounds with various functions, including antiviral compounds; however, high-efficiency methods to screen such compounds are lacking. Advanced virtual screening techniques can significantly reduce the time and cost of novel antiviral drug identification. In this study, we used a cyanobacterial secondary metabolite library as an example and trained three models to identify compounds with potential antiviral activity using a machine learning method based on message-passing neural networks. Using this method, 364 potential antiviral compounds were screened from >2000 cyanobacterial secondary metabolites, with amides predominating (area under the receiver operating characteristic curve value: 0.98). To verify the actual effectiveness of the candidate antiviral compounds, HIV virus reverse transcriptase (HIV-1 RT) was selected as a target to evaluate their antiviral potential. Molecular docking experiments demonstrated that candidate compounds, including kororamide, mollamide E, nostopeptolide A3, anachelin-H, and kasumigamide, produced relatively robust non-covalent bonding interactions with the RNase H active site on HIV-1 RT, supporting the effectiveness of the proposed screening model. Our data demonstrate that artificial intelligence-based screening methods are effective tools for mining potential antiviral compounds, which can facilitate the exploration of various natural product libraries.

## 1. Introduction

Human health infrastructure and knowledge related to the control of infectious diseases have progressed significantly. Nevertheless, we have still witnessed an unprecedented explosion of viral epidemics over the past decade, including H1N1 IAV in 2009, MERS-CoV in 2012, EBOV in 2014, ZIKV in 2015, and SARS-CoV-2 in 2019, and as the environment and climate change, more viral epidemics may emerge and re-emerge [1]. To minimize the threat to human health and socioeconomic development posed by the recurrence of large-scale viral outbreaks, rapid and effective methods of antiviral drug development require further investigation and application. Currently, antiviral drug development strategies are mainly based on screening, designing, and developing drugs targeting viruses or host cytokines [2] via three main steps: first, identification of the mechanism of action the target drug, followed by screening for compounds according to the characteristics of the target, and finally modifying the screened compounds based on simulation or experimental results [3]. Virtual drug screening technologies have benefited from the continuous and rapid development of computer hardware, software, and algorithms, which have greatly reduced the time and expense involved in drug development [4].

Mainstream virtual screening approaches currently applied to antiviral drug development include molecular docking as well as machine learning methods. Molecular docking methods are arithmetic-intensive and relatively time-consuming because they use 3D molecular structures for conformational simulation and force field analysis, meaning that additional criteria are often required to screen large numbers of molecules in the first step (e.g., Lipinski’s Rule of Five [5]) prior to performing molecular docking experiments. Machine learning methods can become skilled at prediction of highly abstract molecular features, which can save time relative to molecular docking techniques, and are suitable for large-scale virtual screening of molecules. Gawriljuk et al. evaluated the performance of several machine learning algorithms (random forest, support vector machine, k-nearest neighbor, Bernoulli naïve Bayes), using extended connectivity fingerprints (AdaBoost decision tree and three-layer deep neural network) as descriptors in classifying new compounds with activity against yellow fever virus [6]. Further, Choi et al. used molecular docking and machine learning models to determine that pentagastrin is a potential antiviral drug against African swine fever virus [7], while Perovic et al. applied molecular interaction field descriptors for principal component analysis (PCA) combined with a molecular docking approach to identify drug candidates targeting the influenza A virus non-structural protein, NS1 [8]. Thafar et al. combined several computational techniques, including feature representation learning, graph mining, and machine learning, to build their model, and in particular, they applied a sequence-to-sequence learning model (seq2seq, also known as the encoder-decoder model) to obtain the key properties of each drug, generate features, and predict drug–target binding relationships in the absence of 3D structural data [9]. Moreover, Rajput et al. extracted 300 unique anti-Ebola compounds and their respective semi-inhibitory concentration values from the “DrugRepV” database using robust machine learning techniques comprising support vector machine, random forest, and artificial neural network approaches, followed by regression-based model development based on quantitative conformational relationship data relating to molecules experimentally active against Ebola [10]. Although the studies mentioned above have demonstrated the functionality of machine learning for antiviral drug screening, researchers have focused more strongly on molecular fingerprint data, rather than molecular structure information, in the virtual screening of antiviral drugs using different machine learning methods. Further, while most molecular fingerprints are generated based on molecular structures, it is challenging for models to learn structural features from molecular fingerprints based on physicochemical properties. Mukherjee et al. attempted to extract structural features from molecules using graphical neural networks combined with other methods to predict binding affinity values for interactions between U.S. Food and Drug Administration-approved drugs and SARS-CoV-2 viral proteins [11]. This approach provides new ideas applicable to the development of novel antiviral screening models.

Inspired by the work of Gilmer et al., in this study, we used a message-passing network (MPN) [12], which is a type of graph neural network able to update the feature representations of nodes by passing messages between nodes, to learn the data representation of the entire graph structure. Prior to this study, the MPN approach was applied to screening antibiotics, where Stokes et al. used only 2335 different molecules to train an MPN to identify potential lead compounds with anti-*Escherichia coli* activity among 107 million molecules [13]. In addition, Wong et al. applied graph neural networks to predict the antibiotic activity and cytotoxicity of 12,076,365 compounds, alongside an interpretable graph algorithm to identify the substructure-based fundamental features of compounds with high predicted antibiotic activity and low predicted cytotoxicity [14]. Based on these results, we speculated that such an approach has potential for application in the exploration of antiviral compounds; however, no research in this area has been reported to date. Here, we used a library of cyanobacterial secondary metabolites [15] as an example to screen for potential antiviral molecules. The cyanobacterial compound library was chosen because it includes a diverse range of secondary metabolites (>2000 substances) and their various functions, including anti-inflammatory, antibacterial, and antitumor effects, among others, and for its potential as a source of antiviral drugs. We used an MPN as a molecular feature extractor and evaluated the applicability of cyanobacterial secondary metabolites as antiviral molecules via molecular docking screening. Our ultimate goal was to explore new methods of screening for antiviral compounds.

## 2. Results

**Figure 1:**
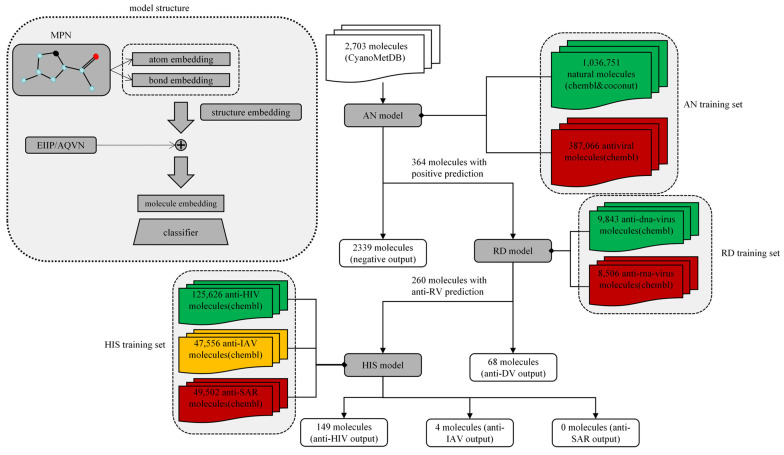
Screening workflow and model structure. The three gray squares represent the AN model for determining the presence or absence of antiviral material, the RD model for assessing tendency to have anti-RNA or -DNA virus activity, and the model for determining activity against the three viruses, HIV, IAV, and SAR. Linking lines indicate the datasets used to train the different models, with arrows showing model inputs and outputs. The gray shaded area in the upper left corner explains the internal structure of the model, in which the message-passing network (MPN) learned and extracted atom embeddings and bond embeddings that contain local physicochemical information from molecules using molecule structures as topology graphs, with atoms as nodes and bonds as edges; the two types of embeddings of molecules were spliced together to obtain the structural embedding of the molecule. The ratio between molecular electron–ion interaction potential (EIIP) and average quasi-valence number (AQVN) was introduced into the structural embedding as a form of per-position summation to finally generate the molecular embedding and input it into the classifier.

In this study, we trained three separate neural network models using an MPN as a molecular feature extractor (Figure 1). The first model was used to look for differences between substances with established antiviral activity and other substances and is referred to here as the antiviral/non-antiviral (AN) model. The second model was applied to find differences in physicochemical characteristics between molecules with antiretroviral activities and molecules with anti-DNA virus function, and we named it the RNA/DNA virus (RD) model. The third model was used to identify differences in the physicochemical characteristics of substances active against three typical RNA viruses, namely, HIV, IAV, and SAR, and is referred to as the HIV/IAV/SAR (HIS) model. Notably, the training and test sets comprised all drugs currently applied as antiviral therapies, represented by their chemical structures obtained from the ChEMBL database. These positive samples facilitated the learning of the underlying principles of molecular feature modelling by our models. The three trained models were applied to a library of cyanobacterial secondary metabolites in a hierarchical screening pattern. We selected relevant receptor targets of HIV and HBV for molecular docking validation of screened molecules.

### 2.1. Neural Network Model Screening of Antiviral Candidate Substances

**Figure 2:**
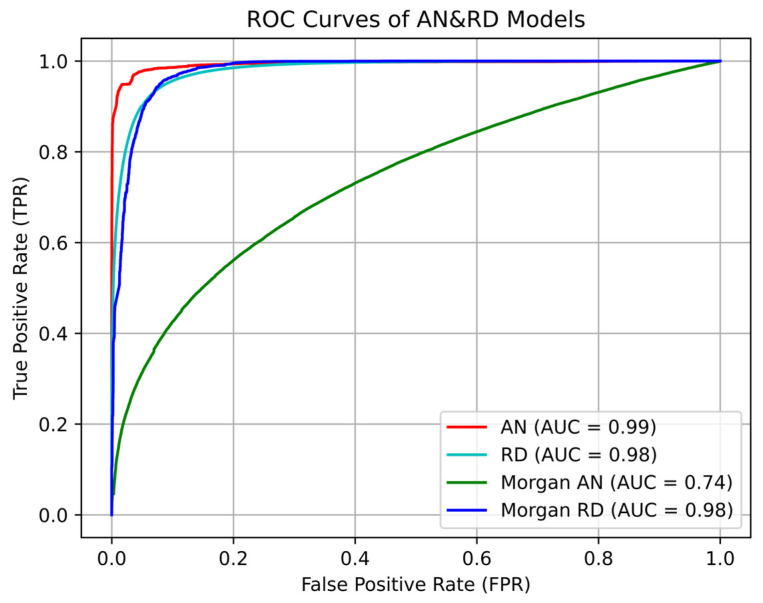
Comparison of receiver operator characteristic (ROC) curves for the AN and RD models using message-passing network (MPN) and Morgan fingerprints: red curve: AN model using an MPN fingerprint; cyan curve: RD model using an MPN fingerprint; blue curve: AN model using a Morgan fingerprint; green curve: RD model using a Morgan fingerprint.

**Figure 3:**
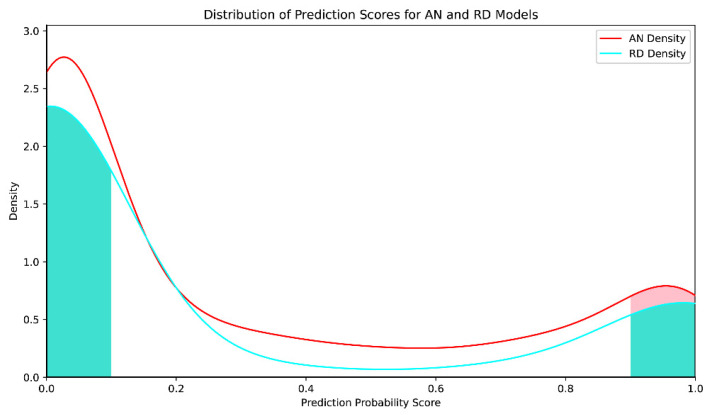
AN and RD models prediction probability score distributions. Horizontal coordinates: prediction probability score of the model’s output. Vertical coordinates: normalized probability density. Red curve: distribution of predicted probability scores from the AN model; red shading: selected antiviral candidates with predicted probability > 90%. Cyan curve: distribution of predicted scores from the RD model; cyan shading (left): set of molecules selected with a predicted probability of anti-DNA virus activity < 10% and probability of anti-RNA virus activity > 90%; cyan shading (right): set of molecules with a predicted probability of anti-DNA virus activity > 90%.

**Figure 4:**
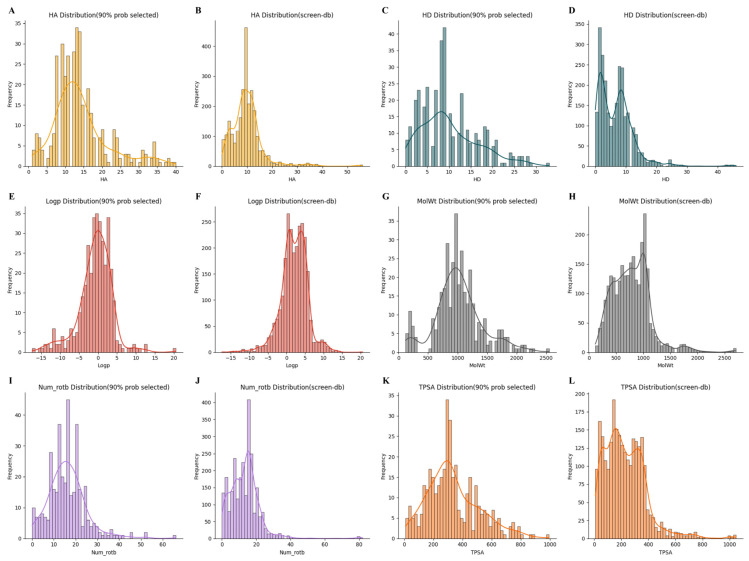
Comparison of the molecular property distributions of cyanobacterial secondary metabolites before and after AN model screening. Distribution plots of the same color indicate comparisons of the same molecular properties. Horizontal coordinates: molecular property metrics. Vertical coordinates: number of molecules. (**A**,**B**) number of hydrogen-bond acceptors; (**C**,**D**) number of hydrogen-bond donors; (**E**,**F**) alcohol–water partition coefficient; (**G**,**H**) relative molecular mass; (**I**,**J**) number of rotatable bonds; (**K**,**L**) topological polar surface area.

AN model training consumed 17 h 8 min, with a maximum area under the receiver operator characteristic (ROC) curve (AUC) value of 0.98 (mean value: 0.959; 95% confidence level error: 0.000627) in five-fold cross-validation (Figure 2). Furthermore, the model demonstrated an accuracy rate > 95% for antiviral drugs commonly employed in therapeutic regimens, including zidovudine and tenofovir. These findings indicate that the model obtained by training had a high classification value and good ability to distinguish the characteristics of antiviral substances from those of general substances. The distribution of probability results indicating whether molecules in the cyanobacterial secondary metabolite library have antiviral activity predicted by the AN model is presented in Figure 3. The prediction results of most molecules were concentrated around 0, while 364 (14.1% of all molecules in the secondary metabolite database) molecules had prediction probability values > 0.9. The distribution of physical and chemical properties of the screened molecules (Figure 4) showed an obvious single-peak compared with pre-screening, with numbers of hydrogen bond acceptors and hydrogen bond donors concentrated around 12 and 9, respectively, the number of molecules with rotatable bonds concentrated around 15, molecular mass was concentrated around 1000 Da, the center of the peak of the alcohol–water separation coefficient being close to 0 (but densely distributed in the interval from 0 to 5), and the topological polar surface area (TPSA) [16] being centrally distributed around 300 Å2, with most molecules with TPSA values < 200 Å2 being discarded.

RD model training consumed 2 h 7 min, and the maximum AUC value in five-fold cross-validation was 0.9987 (mean: 0.997; 95% confidence level error: 0.000631) (Figure 4). The RD model is used to screen the subset of molecules initially screened using the AN model as a library of molecules potentially having antiviral activity. Sixty-eight molecules were predicted as tending to have anti-DNA viral activity (probability > 0.9), representing 18.7% of molecules initially screened using the AN model. The total number of molecules with prediction probability values < 0.1, indicating that they tended to have anti-RNA viral activity, was 260, which was 71.4% of molecules identified via preliminary screening with the AN model. The dichotomous RD model sampled and learned the characteristics of molecules with antiviral activities against three representative RNA viruses, namely, HIV, IAV, and SAR, as well as HBV, as a typical DNA virus, in the training stage, and prediction results were concentrated in a bipartite distribution, indicating that molecules screened by the AN model had the characteristics of molecules with activity against the four types of virus.

We also conducted comparison experiments using Morgan fingerprints, which are currently widely used to describe molecular physicochemical information, as an alternative to molecular fingerprints output by MPNs. In this experiment, the radius of Morgan fingerprints was set as 2 and the number of output bits as 1024, and the same two-layer feed-forward neural network was used as the classifier as that used for the AN and RD models. Finally, ROC curves of the classifier using Morgan fingerprints as molecular features were obtained (Figure 2), and they revealed that Morgan fingerprints performed significantly worse than the molecular feature fingerprints extracted using the MPN in distinguishing whether a molecule had antiviral properties or not (AN model), but they did not perform significantly differently in distinguishing between molecules with activities against RNA or DNA viruses (RD model). This phenomenon may indicate that Morgan fingerprints are more useful for characterizing the local features of molecules; that is, Morgan fingerprints may be better at characterizing local structural information of molecules, where the scope of the local definition is determined by the radius size set by the researcher. In contrast, the MPN can flexibly scale the size of the topology of interest according to the purpose of the task and thus achieved good performance in both the AN and RD models.

**Figure 5:**
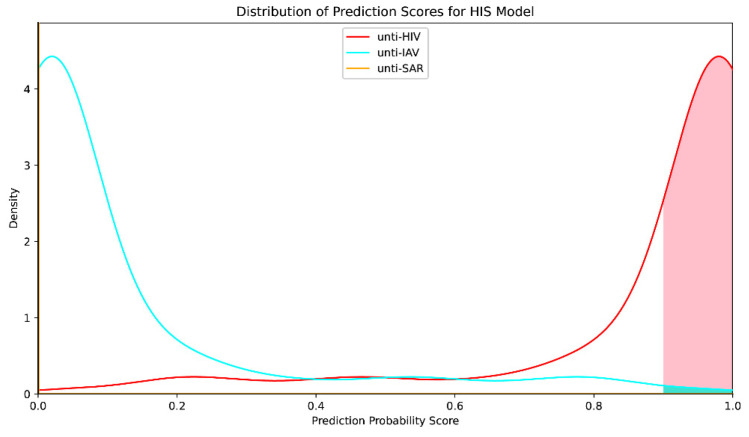
HIS model prediction probability score distributions. Red, cyan, and yellow curves: distributions of anti-HIV, anti-IAV, and anti-SAR virus predicted probability scores, respectively. Shaded portions: selected molecules with predicted probability > 90%.

To further test the ability of the models to discriminate the characteristics of molecules with activities against specific viral species, the HIS model was trained and applied. In contrast to the AN and RD models, the HIS model is a multiclassification model for which ROC curves could not be plotted to obtain an AUC metric encompassing the three classes. To assess the capacity of the HIS model to discriminate among substances targeting three different viruses, the cross-entropy metric was employed. HIS model training consumed 19 h 15 min, and the minimum cross entropy metric in five-fold cross-validation was 0.219 (mean: 0.227; 95% confidence level error: 0.004357). The HIS model was applied to predict the activity of 260 molecules with positive prediction results < 0.1 against the three RNA viruses using the RD model (Figure 5). The HIS model scored each molecule three times in the prediction, representing the likelihoods of prediction of anti-HIV, anti-IAV, and anti-SAR activities. In total, 149 (5.5% of all molecules in the cyanobacterial secondary metabolite library), 4 (0.15% of secondary metabolites), and almost 0 molecules were found to have significant likelihoods (>0.99) of anti-HIV, anti-IAV, and anti-SAR virus activities, respectively.

### 2.2. Statistical Analysis

**Figure 6:**
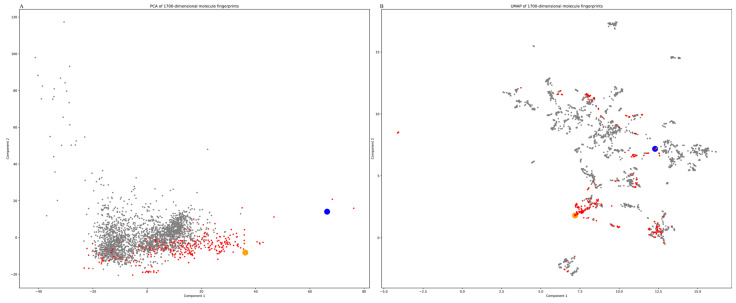
MPN feature extraction fingerprint dimensionality reduction analysis. (**A**) Linear principal component analysis (PCA) dimension reduction. (**B**) Uniform manifold approximation and projection (UMAP) dimension reduction. Scattered dots represent the 2703 molecules in the cyanobacterial secondary metabolite library; red dots: positive outputs from the AN model; blue and yellow dots: kororamide and nostopeptolide A3, respectively, which were used to perform molecular docking validation analysis.

The results of PCA of the 2703 molecules in the cyanobacteria secondary metabolite library indicated that the first two principal components accounted for 9.9% and 6.4% of the total variance. Considering that the molecular fingerprint features output by the feature extraction network comprised 1700 dimensions and that the third principal component only occupied < 3% of the total variance, the first two principal components can be used to demonstrate the effects of model feature extraction. In Figure 6A, horizontal and vertical coordinates indicate the values of the first and second principal components, respectively, while the scattered dots indicate the 2703 molecules in the CyanoMetDB database; gray dots represent molecules with negative AN model output, while red dots indicate those with positive output. Molecules predicted to have antiviral potential are mainly clustered in the lower right part of the first quadrant, and there is a clear demarcation between them and negatively predicted molecules, suggesting that the molecular features extracted using the MPN are able to explain why the molecules may have antiviral properties under linear combination conditions. The data presented in Figure 6B represent the optimal dimensionality reduction distribution of the nonlinear variations of molecular fingerprints under the uniform manifold approximation and projection (UMAP) condition, where the results of streaming analysis correspond more closely to the real distances between molecular fingerprints than those of Euclidean distances in high-dimensional space. Positive predicted molecules exhibited a polycentric distribution, suggesting that at least five classes of similar substances among cyanobacteria secondary metabolites may possess antiviral properties. The larger yellow and blue dots in Figure 6B represent the molecules, kororamide and nostopeptolide A3, which are predicted antiviral substances in the amide and peptidyl lactone categories, respectively, and are discussed in Section 2.3 as validation subjects for molecular docking analysis.

### 2.3. Molecular Docking Study

The HIV reverse transcriptase (HIV-1 RT), RNase H [17], was selected as a receptor protein for molecular docking validation of anti-HIV viral candidates obtained from screening using the HIS model. HIV-1 RT RNase H has a key role in the reverse transcription process of the HIV virus, functioning to hydrolyze viral RNA templates to provide templates for DNA synthesis. Absence of RNase H activity leads to defects in the reverse transcription process, producing incomplete viral DNA, which affects the infection ability of the virus. Thus, viral replication and infection can be prevented by molecules that compete for the RNase H active site.

**Figure 7:**
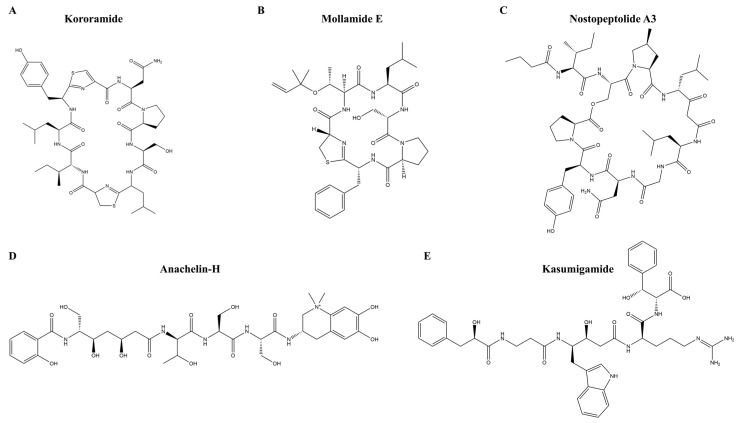
Molecular structures of five potential antiviral compounds. (**A**) kororamide; (**B**) mollamide E; (**C**) nostopeptolide A3; (**D**) anachelin-H; (**E**) kasumigamide.

We performed docking experiments on the 149 molecules obtained by screening using the HIS model, the vast majority of which could generate polar interactions at the HIV-1 RT RNase H active site, with 97 molecules having calculated binding energies < −5 kcal/mol. We selected five representative molecules that formed non-covalent bond interactions with the HIV-1 RT RNase H binding pocket for further analysis (Table 1). Structures of the five molecules are illustrated in Figure 7.

**Figure 8:**
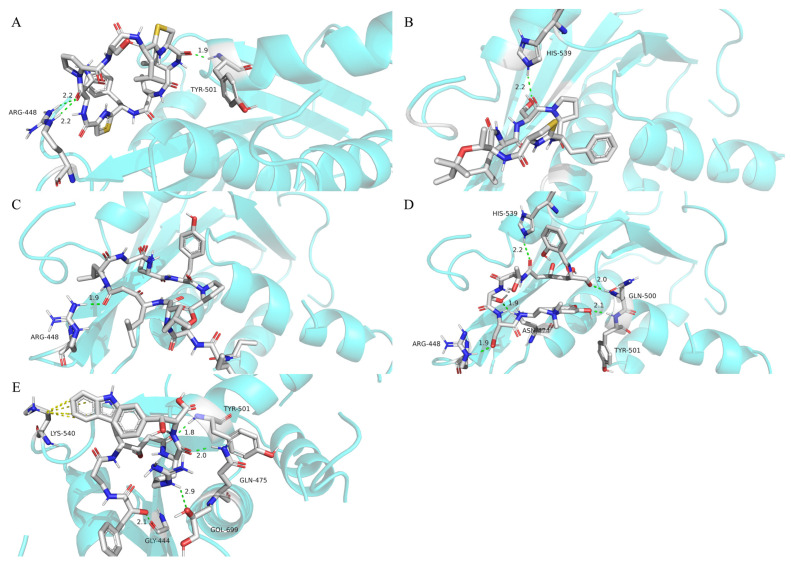
Interactions between the active binding site of HIV reverse transcriptase and five different molecules. Cyan models: proteins; green discontinuities: hydrogen bonding between small molecules and proteins; yellow cones: pi–cation activity. The sub-icon numbers and corresponding numerators are as follows: (**A**) kororamide; (**B**) mollamide E; (**C**) nostopeptolide A3; (**D**) anachelin-H; (**E**) kasumigamide.

Kororamide [18] is a cyclic amide with molecular mass 969 g/mol [19] that forms three hydrogen bonds with two hydrogen atoms on the linked guanidinium nitrogen atom of amino acid ARG448, and a hydrogen atom on the main chain of amino acid, TYR501, in HIV-1 RT RNase H, with a total energy of −8.1 kcal/mol (Figure 8A). Mollamide E [20] is a cyclic peptide conjugate with molecular mass 698 g/mol that forms one hydrogen bond with a hydrogen atom in HIV-1 RT RNase H amino acid HIS539, with a bonding energy of −7.4 kcal/mol (Figure 8B). Nostopeptolide A3 is a cyclic peptide with molecular mass 1081 g/mol that forms a hydrogen bond with a hydrogen atom on the hydrogen-linked guanidinium nitrogen atom in amino acid ARG448 of HIV-1 RT RNase H, with a binding energy of −7.3 kcal/mol (Figure 8C); notably, this hydrogen atom is the same as one of those that forms a hydrogen bond with the kororamide molecule, suggesting that there are local similarities between nostopeptolide A3 and kororamide. Anachelin-H is a linear iron-carrier molecule produced by cyanobacterial cells, with molecular mass 779 g/mol, that forms five hydrogen bonds with hydrogen atoms on amino acids HIS539, GLN500, TYR501, ARG448, and ASN474 in HIV-1 RT RNase H; the hydrogen atoms in ARG448 and HIS539 bound by anachelin-H are the same as those bound by nostopeptolide A3 and mollamide E, respectively. The bonding energy of anachelin-H is −7.1 kcal/mol (Figure 8D). Kasumigamide is a linear polypeptide amide produced by the metabolic processes of cyanobacteria, with molecular mass 786 g/mol, which forms four hydrogen bonds with the amino acids TYR501, GOL669, GLY444, and GLN500 of HIV-1 RT RNase H. The hydrogen atom in the TYR501 amino acid corresponds to that forming hydrogen bonds with anachelin-H. Further, kasumigamide also generates pi–cation interactions with amino acid, LYS540, with a total binding energy of −6.4 kcal/mol (Figure 8E). The results of visual docking analysis indicated that a keto group on kororamide forms two cross-hydrogen bonds with the guanidinium hydrogen atom of ARG448. We postulate that such interactions are most likely attributable to the fact that the keto group forms a specific structure able to compete with its neighboring N-substituted amide for the HIV-1 RT RNase H active site. The keto group was observed to compete with its neighboring N-substituted amide and pyrrolidine rings for the RNase H active site. The same structure, which is involved in a hydrogen bonding interaction, was also identified in mollamide E and the imidazole ring in HIS539.

## 3. Discussion

In this study, we describe the first application of an MPN as a molecular feature extractor in the process of antiviral drug screening. The AUC value of the AN model and the distribution of physicochemical properties before and after the screening demonstrate that the output features of the MPN, together with the molecular electron–ion interaction potential (EIIP)/average quasi-valence number (AQVN) ratio, can effectively differentiate between substances with antiviral activity, which are reported to have antiviral activity in the literature, and natural products in general, or between molecules that do not possess antiviral activity and general natural products or other molecules without antiviral activity. These findings suggest that the molecules with antiviral activity share common features at the atomic structure and atomic charge distribution levels, which can serve as the basis for determining the physicochemical properties of the screened molecules. The data presented in Figure 3 illustrate the distributions of numbers of hydrogen bond acceptors, hydrogen bond donors, rotatable bonds, molecular masses, alcohol–water partition coefficients, and TPSA of molecules screened using the AN model with values > 0.9 (left) and those of the entire library of cyanobacterial secondary metabolites (right). The first five of these physicochemical metrics comprise Lipinski’s rules, which were proposed in 1997 by Christopher A. Lipinski, a senior medicinal chemist at Pfizer, as the basic rules for screening drug-like molecules. These principles are primarily used to assess whether a compound has the potential to become a drug, particularly during the initial screening phase of drug development. The peaks in the distributions of screening results from the AN model exceeded the Lipinski rule limits of molecular weight < 500 g/mol (maximum: 1000 g/mol), hydrogen bond donors < 5 (maximum: 10), hydrogen bond acceptors < 10 (maximum: 11), and the number of rotatable bonds < 10 (maximum: 14); only the peak alcohol–water partition coefficient (close to 0) satisfied the rule (<5). This finding may reflect the fact that the MPN cannot adequately account for the overall size of a molecule when learning the structural features of the atoms within the molecule and is overly interested in the contribution of local structural features to a molecule’s antiviral activity score. If a molecule is too large, it will be difficult for it to cross a biofilm, leaving it unable to bind to its corresponding active site. To reduce the size of screened molecules and to explain the basis of the AN model, we used a structural tree search algorithm, starting from the outer edges of the molecules, to analyze the sub-structures of molecules that had decisive roles in better model predictions. In addition, TPSA correlates significantly with human small intestinal absorption, Caco-2 monolayer permeability and blood-brain barrier penetration. When the TPSA of a monolayer is >140 Å^2^, its penetration in cells becomes poor, which is consistent with the fact that distribution of the remaining physicochemical properties exceeded Lipinski’s rule, and the poor molecular-scale learning of the MPN model. The excellent performance indexes of the RD and HIS models illustrate the superiority of the MPN approach in learning the local structural features of molecules. Eight of the molecules with high anti-HIV prediction scores (among the top twenty) using the HIS model belonged to the cyclic amide class, as follows: kasumigamide, almiramide_E, micromide, tasiamide B, mollamide E, aeruginosamide, 5-epidysidamide G, and tasiamide F. This is a good example of how the HIS model can learn the local structural features of a molecule at the molecular scale. The docking results for both nostopeptolide A3 and kasumigamide indicated the formation of hydrogen bonding interactions by N-substituted amides. These results also elucidated that cyclic amides had higher scores in the model prediction results, indicating the great potential of cyclic amides as anti-HIV viral precursor drugs, which may be related to the solubilizing properties of the amides, as well as their cellular metabolic properties.

The results of molecular docking showed that molecules screened using the HIS model could generate at least moderately strong binding energies with the active binding site of HIV-1 RT RNase H and that different molecules were linked to the same hydrogen atoms in the binding pocket to form hydrogen bonds, where the hydrogen bonds formed with these hydrogen atoms had stronger binding energies (e.g., the molecules presented in Figure 8A,C formed hydrogen bonds with a hydrogen atom of the RNase H ARG448 hydrogen-linked guanidine nitrogen atoms), suggesting the presence of key atoms in the active site of RNase H, which could assist researchers developing new drugs, by improving drug molecular structures in a targeted manner. Indeed, during training of the HIS model, we not only used active molecules targeting HIV-1 RT RNase H as learning samples but also collected 21 molecules targeting proteins that play key roles in the HIV infection replication process, including the envelope glycoprotein, gp160, PBS RNA, and aberrant vpr protein. The results demonstrated that the HIS model has good ability to learn the common features in molecules active against these different target proteins and suggest a new scheme for the binding conformation and key binding sites of the HIV-1 RT RNase H binding pocket.

## 4. Materials and Methods

### 4.1. Dataset Establishment

All cyanobacterial secondary metabolites in the target screening library were downloaded from the CyanoMetDB database, which contains structural information, comprising simplified molecular input line entry system (SMILES) codes, for 2703 products from various sources that have been reported in the literature. The chemicals in the screening library include linear peptides, cyclic peptides, and non-peptide small molecules, with molecular masses mainly distributed in the range of 200–2000 g/mol.

The dataset used for the training and testing of the AN screening model comprised two types of labeled data: Label 0 represented chemical substances that do not have antiviral activity, or natural products that have not yet been found to have obvious antiviral effects, and included 629,481 SMILES codes of chemical substances that do not possess antiviral activity (molecular mass range, 200–2000 g/mol) downloaded from the ChEMBL database and 407,270 SMILES codes of natural products downloaded from the coconut database; and Label 1 represented chemicals that have been reported in the literature and experimentally verified as possessing antiviral activity, including substances with bio-inhibitory activity against viruses such as HIV, SAR, and IAV, comprising 387,066 SMILES codes downloaded from the ChEMBL database. The training set included 1,423,817 SMILES codes tagged with corresponding labels. The primary objectives of constructing the training set in this manner were twofold: first, to fully utilize natural products as negative samples, thereby enhancing the model’s complexity; and second, to ensure an adequate number of negative samples were included in the training set to reflect the actual ratio of negative to positive samples in real-world scenarios, without exceeding a moderate imbalance, and to compel the model to discern the common characteristics of positive samples. The dataset used for training and testing of the RD classification model comprised two types of label: Label 0 denoted anti-HIV viral activity and included 9843 molecules reported in articles within the last five years that showed significant inhibition of HIV viral bioprocesses (EC50 or IC50 ≤ 10 µM); Label 1 indicated anti-HBV viral activity and comprised 8506 molecules. The dataset used for training and testing the HIS classification model comprised three types of labeled data: Label 0 represented 125,626 molecules with anti-HIV-1 activity; Label 1 included 47,556 molecules with anti-IAV viral activity; and Label 2 comprised 49,502 molecules with anti-SAR viral activity. The molecular SMILES chemical formulae and related information in the above three datasets were downloaded from the ChEMBL database.

### 4.2. Screening of Antiviral Candidate Substances Based on the Chemprop Neural Network Model

#### 4.2.1. Model Structure

The molecular antiviral signature screening model used in this study was constructed based on Chemprop software (version v2.0) [21] and modified to improve its suitability for molecular antiviral screening tasks. The prediction model takes the SMILES chemical formula of a substance and the label value as its original input, and its structure was divided into three main parts:

1. An MPN, with atoms as nodes and interatomic bonds as edges, was used to extract features from the original feature expression data, capturing the topological relationships and local structural physicochemical information within molecules. Atomic mass, number of bonds, charge, chiral information, number of hydrogen bonds, hybridization, and atomic mass of each atom in a molecule were obtained as node information using rdkit software (version 2023.03.2). Edge data were obtained based on the type of chemical bond to determine whether bonds were conjugated double bonds, ring bonds, or cis- or trans-double bonds. Further, the rdkit tool was used to transform SMILES chemical formulae into 2D molecular images, using the D-MPNN network to perform directional message passing on the graph, and finally obtain the physicochemical structural characteristics of the molecule. The specific workflow of the MPN was as follows:
  (i) Data of each atomic node and neighboring bond edge were spliced into a vector containing data on only one node and one neighboring edge; for ease of presentation, we refer to this vector as the combination vector, v.
  (ii) The combination vector, v, was multiplied by a learnt parameter matrix, W, to perform the first step coordinate transformation and obtain v1.
  (iii) Other combinatorial vectors adjacent to v1 were weighted and summed, and then added to v1 to obtain v2, where the weight matrix was a learnt parameter. Subsequently, the output vector, v3, was obtained by inputting v2 into the ReLU activation function, and this vector was then used to replace the initial v1.
  (iv) The combined vectors involved in each atomic node were weighted and summed to obtain the atomic (node) feature vector, p, where the weighting parameters were also learnt.
  (v) Finally, all p vectors were weighted and summed, to obtain the molecular feature vector, m; this process refers to the method used in Chemprop.

2. The molecular EIIP/AQVN ratio is believed to explain the unique physical features that define the long-range interactions between biomolecules [22] and was spliced at the tail of the MPN output to form the structural and interaction features of each molecule. EIIP/AQVN can be used to elucidate the distinctive characteristics of long-range interactions between molecules [22] and is robustly correlated with mutagenicity, carcinogenicity, toxicity, antibiotic and cytostatic activity, and other biochemical properties [23]. Moreover, initial research has indicated that molecules with comparable AQVN/EIIP values tend to interact with analogous targets [24]. The reliability of the EIIP/AQVN ratio as a criterion for antiviral drug screening has been validated by several studies [22,25,26], which have demonstrated its efficacy in measuring specific molecule-target interactions. The objective of introducing EIIP/AQVN in the context of molecular characterization was to enhance the classification performance of the model by leveraging prior knowledge. The EIIP/AQVN method predicts the antiviral activity of a molecule mainly based on its electronic properties, which may not be fully reflected by the complexity of the molecular structure, such as three-dimensional conformation, spatial arrangement, and intramolecular and intermolecular interactions. EIIP/AQVN was calculated using the following formula:

(1) $$\mathrm{AQVN}=\sum_{i=1,m}{(n}_{i}Z_{i}/N),$$

(2) EIIP=0.25AQVN sin(1.04πAQVN)

where Z_i_ is the valence number of the i-th atomic component, n_i_ is the number of atoms of the i-th component, m is the number of atomic components in the molecule, and N is the total number of atoms.

3. A feed-forward neural network updated at the same time as the D-MPNN was used to classify the extracted features decoder, whose depth was defined using shortcut commands during training.

#### 4.2.2. Network Training and Testing

The Chemprop package was downloaded and the relevant environment configured in anaconda. The AN model used an MPN with three layers of three for molecule feature extraction. The number of feed-forward neural network layers was set to 2, and the number of hidden layer nodes was set to 300. The dataset was randomly divided into training, validation, and test sets at a ratio of 8:1:1, and training results were evaluated using 5-fold cross-validation. The number of training rounds (epoch) was 30, batch size = 200, binary cross-entropy was used as the loss function, and AUC was used as the model evaluation metric. Given the analogous classification challenges encountered by the classifiers when assessing the performance of Morgan fingerprints and molecular feature fingerprints in this study, as well as the comparable volume of data, the Morgan fingerprint classifier was trained using identical training parameters to those used for the AN model, thereby facilitating more expeditious attainment of optimal training efficacy. The RD model used an MPN with a layer number of 4 for molecule feature extraction; the number of feed-forward neural network layers was set as 2, batch size was set as 20, and a dataset partitioning method was used. The model evaluation index and other parameters were the same as those for the AN model. The HIS model used an MPN with a layer number of 5 for molecule feature extraction, the number of feed-forward neural network layers was set to batch size = 50, and cross entropy was used as the model evaluation index.

Training and testing of the MPN, as well as the feedforward neural network, were performed on a single 24 GB graphics memory NVIDIA A10 GPU, with a 3.4 GHz CPU (Intel Xeon Cooper Lake).

The trained AN model was applied to the target screening library, normalizing the output of the model in the range of [0, 1], to indicate the likelihood of a substance having antiviral activity; the screening threshold was set to 0.9. The trained RD model was applied to the library of suspected antiviral active substances obtained by screening using the AN model (threshold value, 0.9), and the range of output results standardized [0, 1] to indicate the bias of active substances against RNA or DNA viruses, where values closer to 0 indicate more bias toward anti-RNA virus activity. The standardized output result range of the model was set to >0.9 and <0.1. The trained HIS model was applied on the library of molecules with anti-RNA virus activity screened using the RD model (threshold score, <0.1), and output was a normalized [0, 1] predicted activity probability for HIV, IAV, and SAR viruses, where the sum of the three probabilities equaled one.

Structural interpretation of screened antiviral activity candidates was performed using a tree search method to select the smallest substructures that could be recognized by the model as having antiviral activity, setting substructures as consisting of a minimum of 8 atoms and a maximum of 20 atoms.

#### 4.2.3. Statistical Analysis

The solubility coefficient, molecular mass, number of hydrogen bond donors and acceptors, and TPSA of the target screening library and molecules with >90% predicted probability of antiviral activity were enumerated using the RDKIT molecular descriptor calculation tool, and distribution histograms were plotted for comparative analysis. State values of hidden nodes in the feed-forward neural network of the model were extracted to serve as molecular fingerprints, and the molecular fingerprints corresponding to the 2703 molecules in the screening library were subjected to PCA [27]: The n_components parameter of PCA was set to 2. The auto-singular value decomposition method in the Python software (version 3.13.0) library, sklearn [28], was used to perform linear dimensionality reduction of fingerprints, and the analysis results are displayed in the form of a two-dimensional scatter plot, which was used to express the classification characteristics of the molecular fingerprints linearly. The prediction results of the model were further interpreted using the UMAP [29] streaming analysis technique: the n_components parameter was set to 2, and the Python software library, sklearn, was also used for implementation to display the streaming distributions in the form of a two-dimensional scatterplot, which represented the classification properties of the nonlinear transformation of molecular fingerprints and could thus be used to distinguish different molecular clusters.

### 4.3. Molecular Docking Study

Molecular docking validation experiments were conducted using functional structural protein molecules from the HIV and HBV viruses, along with the candidates obtained by model screening; some of these molecules already had accurate 3D models, and the remaining small molecule structures were predicted using rdkit, while large molecule structures were predicted using alphaflod [30]. Eight targets that have been widely used for molecular docking to test affinity activity, including the abnormal vpr protein of HIV, the gp160 encapsidated protein, and protein hydrolases, were selected. To conduct molecular docking studies on 68 substances with potential anti-DNA virus activity, as identified by RD modelling, the core antigen and DNA polymerase of HBV virus were selected as targets for testing affinity activity. The pdb files of receptor proteins were downloaded from the Protein Data Bank database. Discovery Studio visualization software was then used to remove the small molecules originally located in the binding sites in the database, as well as the water molecules surrounding the proteins. Subsequently, hydrogen atoms were added to the protein molecules, which were then saved as pdbqt files. A subset of the structure files for small molecule ligands was obtained directly from the PubChem database. A Merck molecular force field was employed in RDKit’s Python package to perform structural optimization for a portion of molecules for which structural models had not yet been published. To obtain the lowest-energy molecular conformation, small molecules were then subjected to hydrogen addition using Autodock software (version 4.2.6). The center of rotation and number of rotatable bonds were computed and the resulting data saved as a PDBQT file. Molecular docking experiments were performed on the autodock4 platform, using the high-precision computational core, Vina, to calculate ligand-target binding energy. The centre coordinates of the grid box have been established at (5.207, 53.715, 17.505), while the box size have been set at (45.0, 47.25, 47.25). 

## 5. Conclusions

Computational methods have been significant in the discovery of available antiviral drugs based on chemically diversity. Here, we found that incorporation of a machine learning approach accelerated the process of antiviral agent discovery. We applied an MPN approach to develop a robust prediction algorithm, including three models: AN, RD, and HIS. Target compounds screened from the cyanobacterial metabolite database were further confirmed using molecular docking experiments. These methods will help predict true target structures and identify potential antiviral drugs. In addition, the machine learning approach used here can be applied to other databases, such as those derived from plants, fungi, and bacteria, in the future, which could assist in the discovery of broad-spectrum antivirals. Future research is required to test the in vivo efficacy of these substances in cell or animal models infected with viruses.

## Figures and Tables

**Table 1:** Molecular docking results table. The table illustrates the binding energy of five molecules to the RNase H site.

Molecule Name	Affinity (kcal/mol)
Kororamide	−8.1
Mollamide E	−7.4
Nostopeptolide A3	−7.3
Anachelin-H	−7.0
Kasumigamide	−6.4

## Data Availability

The data presented in this study are available on request from the corresponding author.
